# Chain-mediated effects of servant leadership on the physical health of Chinese elementary school teachers

**DOI:** 10.3389/fpubh.2025.1691696

**Published:** 2025-12-03

**Authors:** Hongchao Wu, Ruiqi Zhou, Xiuhong Li, Lihui Yan, Congying Ma

**Affiliations:** 1 School of Education, South China Normal University, Guangzhou, China; 2 School of Education, Kashi University, Kashi, China; 3 School of Art and Design, Guangdong University of Technology, Guangzhou, China; 4 School of Educational Science, Lingnan Normal University, Zhanjiang, China; 5 School of Education, Beijing Institute of Technology, Beijing, China

**Keywords:** servant leadership, physical health, hindrance stressors, emotional exhaustion, elementary teachers

## Abstract

**Introduction:**

The role of servant leadership in the processes of fostering a healthy work environment and enhancing employee well-being has become an important area of academic inquiry, particularly with respect to the highly stressful and emotional demanding profession of teaching. However, the underlying mechanisms of the relationships to teacher’s physical health remain insufficiently understood. Thus, in light of the direct effect hypothesis of social support theory and the job demandsresources model, this study conducted a cross-sectional survey of 827 primary school teachers in China.

**Methods:**

Descriptive statistical analysis was conducted using SPSS 26.0, chain mediation effects were tested using SPSS PROCESS 4.1 (Model 6) with 5,000 bootstrap samples, and the fit of the hypothesized model was further evaluated using AMOS 26.0.

**Results:**

The results revealed significant correlations among servant leadership, hindrance stress, emotional exhaustion, and physical health. Specifically, servant leadership was revealed to be significantly negatively associated with physical health problems. Three distinct mediation pathways were identified: (1) the direct mediating effect of hindrance stress, (2) the direct mediating effect of emotional exhaustion, and (3) the chain-mediated effect of hindrance stress on physical health through emotional exhaustion.

**Discussion:**

These results provide empirical evidence concerning the mechanisms through which leadership influences teachers’ health and contribute to improving the physical health of Chinese elementary school teachers.

## 1 Introduction

The United Nations’ fourth sustainable development goal (SDG 4) focuses on advancing inclusive, equitable, and high-quality education. Achieving this goal is inherently linked to the well-being of teachers, both physically and mentally (1). However, employment across various professional fields can have adverse effects on workers’ well-being (2). Teaching is acknowledged as a high-risk profession because of its demanding nature, significant challenges, and elevated levels of occupational stress (3). An increasing body of research indicates that compared with practitioners in other industries, teachers tend to exhibit poorer physical health (4–6). Worryingly, the data indicate a continuing decline in teachers’ overall health (7). These trends are likely to have far-reaching consequences—not only do they diminish teachers’ capacity and motivation, but they also contribute to increased absenteeism, presenteeism, and teacher attrition, ultimately compromising the quality of education in schools (8).

Enhancing teachers’ well-being has become a critical priority for educational institutions, policymakers, and researchers worldwide. In China, the government has introduced policies aimed at alleviating teachers’ workload pressure and has implemented a legally mandated medical examination system to monitor their health. Research has consistently demonstrated that job stress, burnout, inadequate job resources, and excessive job demands negatively affect employees’ physical well-being (9–11). These findings are equally applicable to the teaching profession (8, 12–14). Notably, primary school teachers experience particularly intense workplace pressure (15), likely because of the demanding nature of working with younger students, who require intensive supervision and support. As a result, primary school teachers must invest more time and energy in their work (16), leading to increased job demands that may ultimately compromise their physical health.

Existing evidence suggests that leadership style is a cornerstone in shaping employees’ health (17–19). Given that teachers’ physical health is fundamental to their overall well-being, understanding the impact of leadership on teachers’ health is essential. A systematic review revealed that leadership is a key contextual factor affecting teacher well-being in the workplace (20). However, educational leaders often prioritize student health and academic achievement while overlooking the well-being of teachers (21). In China, with ongoing societal development and educational reform, many schools have adopted corporate-style management models, such as performance-based pay, which tend to emphasize efficiency and accountability over humanistic care for teachers (22).

Recently, servant leadership has received significant attention in educational settings, emphasizing the holistic development of followers and prioritizing their welfare and progress (23). The notion of servant leadership involves multifaceted engagement with followers across relational, ethical, emotional, and spiritual dimensions, ultimately enabling individuals to realize their greatest potential. Eva et al. (24) define servant leadership as a leadership style focused on the specific needs and interests of followers, shifting their focus from self-interest to the welfare of others within the organization and the wider community. Unlike traditional leadership models that emphasize organizational and individual achievements, servant leadership is fundamentally committed to fostering the holistic development of its followers (25).

Extensive research has demonstrated that servant leadership has a positive impact on followers, primarily in the areas of attitudes, behaviors, and performance-related outcomes (26–31). Studies on servant leadership have likewise expanded into the educational domain. For instance, research involving 620 basic education teachers found that servant leadership enhances both resource satisfaction and life satisfaction (32). Another study involving 1,120 teachers revealed that servant leadership indirectly affects teachers’ emotional commitment and psychological well-being (33). Research examining the impact of servant leadership on teachers’ physical health remains relatively scarce (34). Existing studies have largely approached this relationship from indirect or macro-level perspectives, offering limited empirical support for a direct link between servant leadership and teachers’ physical health. In particular, investigations focusing specifically on primary school teachers are rare, and quantitative studies addressing this relationship are especially limited. Consequently, our understanding of the direct effects of servant leadership on teachers’ physical health remains inadequate, underscoring the need for further empirical inquiry.

Furthermore, research on the mediating mechanisms linking servant leadership to physical health remains in its early stages. Existing studies suggest that servant leadership is closely linked to employees’ stress and burnout (35, 36), both of which, in turn, have considerable ramifications for physical health. Based on this evidence, we propose that stress and burnout may mediate the link between servant leadership and employees’ physical health (37). We specifically examine hindrance stress, a critical aspect of job stress, and emotional exhaustion, a fundamental component of burnout, as two potential mediating variables. Servant leaders generally demonstrate four essential behaviors: empowering followers, encouraging them, offering emotional support, and exercising stewardship (38), which also increases the likelihood that servant leadership may influence teachers’ physical health by reducing hindrance stress and emotional exhaustion. Although empirical research on this topic remains limited, existing evidence suggests that servant leadership can significantly alleviate both emotional exhaustion and hindrance stress, while these two factors exert negative effects on individuals’ physical health (35–37). However, the specific mediating mechanisms through which hindrance stress and emotional exhaustion link servant leadership to physical health have yet to be empirically verified.

According to the job demands–resources (JD-R) paradigm, high job demands diminish individuals’ physical, emotional, and psychological resources, resulting in increased tiredness (39). These findings suggest that hindrance stress may also contribute to emotional exhaustion, resulting in a sequential mediating effect. Despite growing interest in servant leadership within educational settings, most existing studies have predominantly focused on teachers’ psychological well-being, whereas its impact on teachers’ physical health has received limited empirical attention (32, 33). Furthermore, the underlying mechanisms—particularly the mediating roles of hindrance stress and emotional exhaustion—in the association between servant leadership and teachers’ physical health remain underexplored. Notably, empirical evidence on servant leadership among Chinese elementary school teachers remains scarce, highlighting the need for further investigation in this context. Accordingly, this study systematically reviewed relevant concepts and existing literature to formulate research hypotheses and, on this basis, constructed and empirically tested a chain mediation model to examine the relationship between servant leadership and teachers’ physical health. It further sought to clarify the mediating roles of hindrance stress and emotional exhaustion, thereby providing practical strategies for enhancing the physical health of Chinese elementary school teachers.

This research contributes to the literature in three key ways. First, it advances our theoretical understanding by exploring the impact of servant leadership on teachers’ physical health, an area that has received limited attention. By addressing this gap, this study bridges the theoretical connection between servant leadership and followers’ physical health. Additionally, by applying the servant leadership framework to health management, this research introduces a novel theoretical perspective, offering valuable insights for future studies. Second, this research contributes to the literature on the mediating mechanisms between servant leadership and physical health. In this study, a chain mediation model is proposed, with hindrance stress and emotional exhaustion identified as key mediating variables. By offering a systematic analytical framework, this research enhances the understanding of the underlying mechanisms of servant leadership and provides a foundation for future studies to explore the most effective pathways for improving teachers’ physical health. Third, this study provides empirical support for servant leadership among Chinese elementary school teachers, expanding the cultural applicability of this methodology and demonstrating practical significance. Investigations of how servant leadership practices can improve Chinese teachers’ physical well-being yield results that school administrators can use to establish actionable strategies to cultivate a more healthful and sustainable workplace.

The paper is structured as follows. Section 2 reviews the related literature and research hypotheses. Section 3 provides an overview of the selection of participants, variables, and methods. Section 4 presents the empirical results. In Section 5, the results, research implications and limitations and future work are discussed. Section 6 contains the conclusions.

## 2 Theory and hypotheses

### 2.1 Servant leadership and teacher physical health

Servant leadership is defined as “an understanding and practice of leadership that places the good of those led over the self-interest of the leader, emphasizing leader behaviors that focus on follower development while de-emphasizing the glorification of the leader” (40). A defining characteristic of this type of leadership is the servant role, which prioritizes the growth and well-being of others. Given this core attribute, the educational field presents an ideal environment for servant leadership, where leaders support and develop their subordinates not for personal gain (41). This study adopts the conceptualization of servant leadership proposed by Sendjaya et al., which comprises six key dimensions: willing submission, which refers to the leader’s readiness to prioritize the needs of others over personal interests; authentic self, which reflects a strong commitment to self-integrity and accountability; covenantal relationships, characterized by deep, personal, and enduring connections among managers and employees, founded on common principles, reciprocal trust, and integrity; ethical responsibility, which involves engaging others in moral reasoning and ethical behavior; transcendent spirituality, which fosters a sense of purpose, guidance, and interpersonal connection; and transformative impact, which emphasizes leaders’ role in the process of supporting others’ personal and professional growth, thereby enabling them to attain their maximum potential (42).

In this study, physical health is conceptualized as psychosomatic responses to stress, such as neck and shoulder pain, indigestion, and sleep disorders (12). Notably, physical health problems here do not include organic diseases or psychosis. This definition conforms to earlier findings emphasizing the impact of occupational stressors on physical well-being within the teaching profession. It is widely recognized that social support can alleviate individual stress and is positively associated with both physical and mental well-being (22). The direct effects hypothesis proposes that significant social support consistently enhances individuals’ health, enabling them to thrive regardless of whether they are experiencing stress (43). In China, where power distance is relatively high, leaders are often perceived more as authoritative supervisors than as supportive figures by teachers (44). With respect to work-related stress, Chinese teachers tend to suppress or conceal their negative emotions rather than seeking support from their leaders (45). Given this cultural context, we propose that the direct effects hypothesis may more accurately reflect the reality of social support and leadership dynamics in Chinese schools.

Considering servant leadership as a form of social support (29), we argued that it has a universally beneficial impact on teachers’ physical health in various ways. First, existing research indicates that servant leadership fosters a positive emotional state among teachers (46), which, in turn, has been linked to improved physical health outcomes (47). Second, servant leadership is founded on the notion that leaders should empower their subordinates, making them more capable, responsible, healthy, wise, and autonomous (48). Given this philosophy, the well-being of followers—including their physical health—is an inherent objective of servant leadership. Qualitative research suggests that servant leaders provide employees who are experiencing health issues with referral letters to appropriate medical professionals and ensure that they receive adequate leave for recovery (49). Furthermore, an empirical study of 509 teachers in Peru demonstrated that servant leadership acts as a crucial determinant of employees’ overall health (50).

However, the role of servant leadership in teachers’ physical fitness remains insufficiently explored. Therefore, this study intends to examine the relationship between servant leadership and teachers’ physical health. By doing so, it not only provides empirical support for the direct effects hypothesis within the Chinese educational context but also offers practical insights for enhancing the welfare of elementary school educators in China. Grounded in this premise, we posit that servant leadership positively influences teachers’ physical health. Consequently, we propose the following hypothesis:

*Hypothesis 1*: Servant leadership is significantly negatively related to the physical health problems among primary school teachers in China.

### 2.2 The mediating role of emotional exhaustion

Burnout is a widely studied topic in occupational health psychology, with research indicating that employees prone to burnout often experience declining job performance and face an elevated risk of severe health complications over time (51). Burnout is characterized by chronic fatigue, emotional detachment, and a diminished sense of professional efficacy. It is typically defined as “a state of exhaustion in which one is cynical about the value of one’s occupation and doubtful of one’s capacity to perform” (52). The core component of burnout, emotional exhaustion, refers to the profound sense of mental and emotional depletion resulting from occupational demands (52). Alarmingly, a comparative study across 36 countries revealed that Chinese educators report peak degrees of emotional exhaustion (53).

As proposed by the direct effect hypothesis in social support theory, servant leadership may influence teachers’ emotional exhaustion through several mechanisms. First, the compassion and empathy demonstrated by servant leaders can help alleviate teachers’ emotional distress (54). Second, servant leadership cultivates a constructive work atmosphere, improving teachers’ well-being and emotional fortitude (55, 56). These theoretical assertions have received empirical validation. For instance, a study involving 220 Chinese nurses revealed that servant leadership markedly diminishes emotional exhaustion (57). Similarly, research has demonstrated that servant leadership mitigates employee burnout (58). Additional empirical evidence reinforcing this viewpoint has also been identified in the context of educational research. A study on Chinese primary school teachers revealed that servant leadership directly enhances teachers’ emotional well-being (46). Similarly, studies examining middle school teachers and young university faculty in China have demonstrated that servant leadership directly enhances teacher well-being (59, 60).

With respect to the correlation between emotional exhaustion and physical health, Kim et al. (61) reported that welfare officers who reported elevated burnout at the outset were more likely to report a greater number of physical health issues throughout the three-year period. These health issues included sleep disruptions, headaches, respiratory ailments, and gastrointestinal disorders. Additionally, Rothmann et al. (62) empirically demonstrated a positive correlation between emotional exhaustion and poor health outcomes. Furthermore, a systematic review of 21 studies involving 5,267 teachers confirmed a connection between teacher burnout and somatic symptoms such as headaches, as well as illnesses such as inflammation (8). Drawing on the preceding discussion, the second hypothesis is formulated as follows:

*Hypothesis 2*: The relationship between servant leadership and physical health is mediated by emotional exhaustion.

### 2.3 The mediating role of hindrance stressors

In accordance with the challenge–hindrance stressor framework (CHSF) proposed by Podsakoff et al. (63), job demands are divided into two types: challenge stressors that promote task accomplishment and personal development and hindrance stressors that impede goal attainment and professional advancement. Hindrance stress refers to work-related stressors that hinder employees’ ability to accomplish tasks or advance in their careers (64). It is characterized by negative effects, a lack of motivational potential, and resource depletion. Common hindrance stressors include role ambiguity, insufficient resources, bureaucratic constraints, interpersonal conflicts, and organizational politics.

Importantly, the seminal research on the CHSF has revealed that stressors categorized as either challenges or hindrances are linked to elevated physical and mental stress. Primary research and meta-analyses using the CHSF generally indicate that both stressors are positively associated with strain outcomes, such as burnout, emotional exhaustion, weariness, irritation, and manifestations of mental, somatic, and psychological distress (65, 66). Prior research has also shown a positive association between hindrance stressors and physical health problems (67, 68).

According to the JD-R framework, each profession entails specific job demands, which, when coupled with limited job resources, can result in negative health consequences (69). Job demands refer to the physical, psychological, social, or organizational aspects of work that require sustained physical or mental effort and skills, leading to specific physiological or psychological costs (70). Examples include intense job pressure, unfavorable working conditions, and emotionally laborious client engagements. Job resources encompass organizational, social, psychological, and physical elements that (a) assist employees in achieving work objectives; (b) buffer the negative effects of job demands and the related mental and physical toll; or (c) facilitate professional flourishing (9). Within the initial health impairment phase of the framework, jobs that are poorly structured or characterized by persistent demands—such as excessive workload or emotional labor—can progressively deplete employees’ psychological and physical reserves, potentially leading to strain outcomes. However, adequate job resources can mitigate these adverse effects by mitigating burnout and enhancing general well-being (39).

Most hindrance stressors correspond to job demands within the JD-R model (9, 71). Additionally, leadership is crucial in influencing both job demands and job resources (71). Within the JD-R framework, effective leadership can enhance job resources to alleviate the adverse effects of job demands on health while also reducing job demands, thereby minimizing health-related strain. For teachers, job demands impact welfare more than do job resources (12). The detrimental effects of job demands on teachers’ well-being are stronger than the protective effects of job resources (72). Rather than alleviating stress, leadership often exacerbates hindrance stress, as teachers perceive leadership-led interventions as counterproductive—given that administrators organizing these initiatives are often seen as sources of stress (73). Moreover, servant leadership interventions can, in some cases, intensify the link between hindrance stressors and emotional exhaustion (74). Moreover, transformational leadership, which shares significant conceptual overlap with servant leadership (75), has been found to reduce job positions (76). Accordingly, servant leadership may also contribute to lowering job demands.

Considering the previous discourse on this topic, we contend that servant leadership may directly reduce hindrance stressors, thereby decreasing employees’ physical problems. Supporting the needs and interests of subordinates is given more importance in servant leadership (77), and leaders who practice servant leadership tend to create a more fair and harmonious work climate, cutting down the sources of hindrance stress at the root. Empirical evidence supports this reasoning. For instance, servant leadership is strongly correlated with organizational citizenship behavior (78) and perceived organizational justice (79). An empirical study with 203 teachers revealed that principals’ servant leadership positively influences teachers’ organizational citizenship behavior (80). Drawing on the preceding discussion, the third hypothesis is formulated as follows:

*Hypothesis 3*: Hindrance stressors mediate the relationship between servant leadership and physical health.

### 2.4 The chain mediating effect of hindrance stressors and emotional exhaustion

Although the literature indicates that hindrance stress and emotional exhaustion influence the correlation between servant leadership and physical well-being independently, these factors may also be interconnected. Meta-analyses of the Challenge–Hindrance Stress Model indicate that hindrance stressors generally have negative effects on individuals’ psychological stress and physical health (66). Among them, nine studies indicate that hindrance stressors have a substantial beneficial influence on stressor burnout. In line with the Cognitive Activation Theory of Stress, when individuals perceive negative outcomes as unavoidable, they may experience helplessness and subsequently develop emotional exhaustion (81). Workplace hindrance stressors, which hinder employee growth and disrupt task completion while depleting energy and resources, may contribute to adverse emotional responses. This can create a sense of inescapability, further exacerbating emotional exhaustion. Xie et al. (81) also identified a direct link between hindrance stressors and emotional exhaustion. Building on this foundation, we posit that servant leadership may mitigate emotional exhaustion by alleviating hindrance stressors, thereby influencing teachers’ physical health. Therefore, we propose the fourth research hypothesis:

*Hypothesis 4*: Hindrance stressors and emotional exhaustion act as chain mediators in the relationship between servant leadership and physical health.

**Figure 1:**
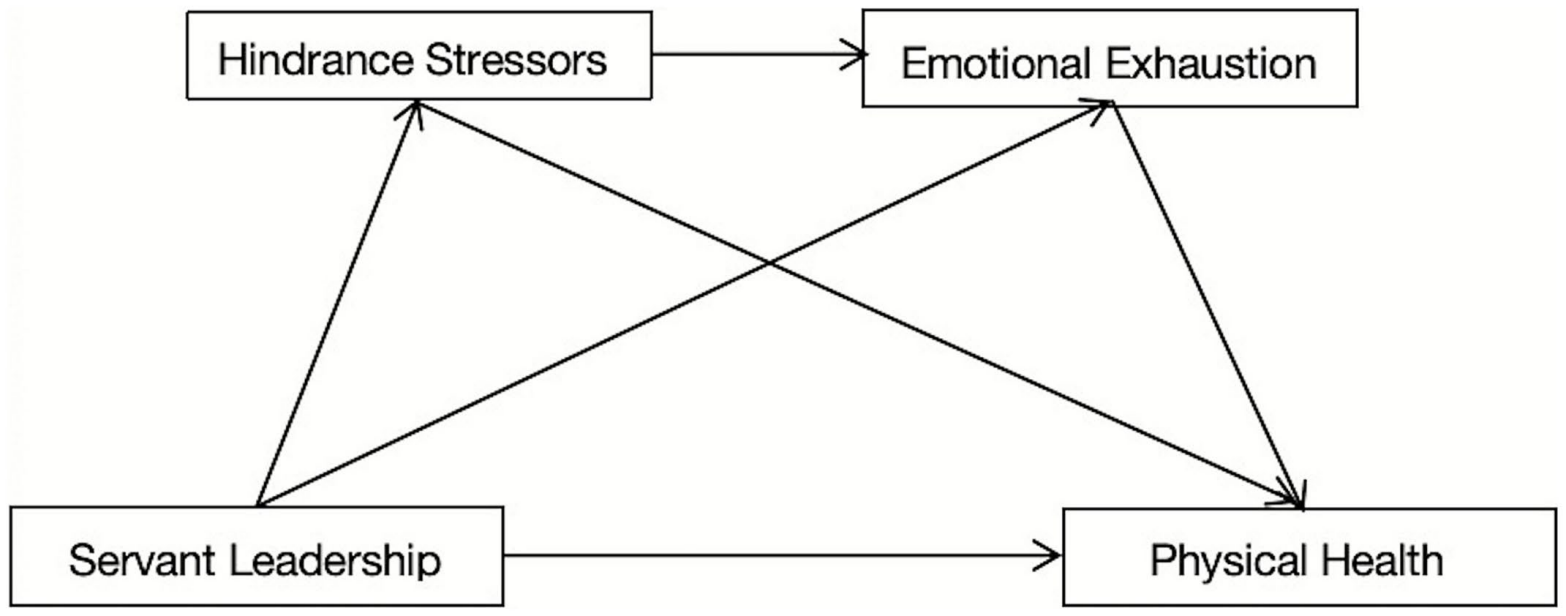
Research hypothesis model diagram.

Building on this framework, this study aims to investigate how servant leadership affects primary school teachers’ physical health and clarify how emotional exhaustion and hindrance stressors play a chain-mediated role in the relationship between servant leadership and physical health. By combining the JD-R model with the direct effects hypothesis of social support theory, this study aims to elucidate how servant leadership affects primary school teachers’ physical health. The proposed chain mediation model is presented in Figure 1.

## 3 Materials and methods

### 3.1 Participants and procedure

This study employed a random sampling method to select regular primary school teachers in South China who did not hold leadership positions such as subject leaders, grade leaders, or directors. A total of 1,016 questionnaires were collected, with 827 valid responses retained after exclusions, yielding an efficiency rate of 81%. The sample included 121 male teachers (14.6%) and 706 female teachers (85.4%), with 681 teachers from public schools (82.3%) and 146 from private schools (17.7%). It is noteworthy that the number of male participants was considerably smaller than that of female participants, which can be attributed to the fact that, according to the latest statistics released by the Ministry of Education of the People’s Republic of China, males account for only 25.7% of primary school teachers nationwide (82). The participants’ average age was 34.26 years, with 38.7% aged 20–29, 32.3% aged 30–39, 21% aged 40–49, and 8% aged 50 or above. The average teaching experience was 11.85 years.

Informed consent was obtained from all participants in this survey. The surveys were distributed and gathered via the Questionnaire Star platform, a frequently used professional online survey tool in China (83). It has been highly recognized by users for its efficiency and convenience. The study team offered a reminder to the participants in the questionnaire information, assuring them that the survey was confidential, that the data would simply be employed for research reasons, and that it would have no effect on their professional or personal lives. The survey protocol was approved by the ethics committee and adheres to the principles stipulated in the Declaration of Helsinki.

### 3.2 Measures

#### 3.2.1 Servant leadership

In the current study, the six-item brief version of the Servant Leadership Behavior Scale (SLBS-6) was employed to measure perceptions of servant leadership (84). Elementary teachers, who served as the participants, rated their principals’ behaviors on a five-point Likert scale from 1 (“strongly disagree”) to 5 (“strongly agree”). A sample item from the scale includes the following: “My principal contributes to my personal and professional growth.” Previous research has reported a Cronbach’s *α* of 0.89 for this scale (84). In this research, the Cronbach’s α coefficient was 0.82, while the composite reliability (CR) reached 0.825, both of which surpassed the recommended threshold of 0.7, thereby confirming the high internal consistency of the scale. Additionally, the average variance extracted (AVE) for assessing convergent validity was 0.542, indicating strong consistency among the items representing the latent variable.

#### 3.2.2 Hindrance stressors

Hindrance stressors were assessed using the 10-item scale developed by LePine, Zhang, Crawford, and Rich (85). One example item from this scale is “The conditions for me to complete tasks are insufficient.” Participants assessed the items using a five-point Likert scale ranging from 1 (“strongly disagree”) to 5 (“strongly agree”). Previous research reported a Cronbach’s *α* of 0.93 for this scale (85). In this investigation, the Cronbach’s α reached 0.89, and the composite reliability (CR) was 0.890, both of which exceeded the 0.7 threshold, thus confirming the high internal consistency of the scale. The average variance extracted (AVE) was 0.505, suggesting strong consistency among the items representing the latent variables.

#### 3.2.3 Emotional exhaustion

Emotional exhaustion was measured using a 9-item subscale drawn from the Maslach Burnout Inventory (MBI) (86), which evaluates the extent of emotional strain or fatigue experienced by participants due to work demands. An example item from this subscale is “I feel I have lost my passion for work.” The respondents evaluated the items on a five-point Likert scale ranging from 1 (“strongly disagree”) to 5 (“strongly agree”). Maslach and Jackson (86) previously reported a Cronbach’s *α* of 0.89 for this subscale. In the current study, the Cronbach’s α was 0.91, and the composite reliability (CR) was 0.912, both of which exceeded the recommended threshold of 0.7, thereby confirming the high internal consistency of the scale. The average variance extracted (AVE) was 0.569, indicating strong consistency among the items representing the latent variables.

#### 3.2.4 Physical health

Physical health was assessed on the basis of a five-item scale (12), which encompassed (a) neck, back, or shoulder discomfort; (b) gastrointestinal difficulties; (c) headaches; (d) vertigo; and (e) sleep abnormalities. Participants assessed the extent to which these health conditions impacted them over the previous academic year on a four-point scale, ranging from “not at all bothered” (1) to “very much bothered” (4). In the current study, the Cronbach’s *α* was 0.84, and the composite reliability (CR) was 0.842, both of which exceeded the 0.7 benchmark, confirming that the scale has strong internal consistency. The average variance extracted (AVE) was 0.521, indicating that the items that encode the latent variables were very consistent with each other.

### 3.3 Data analysis

The results of the survey were analyzed using both SPSS 26.0 and AMOS 26.0. First, we evaluated the reliability and validity of the measurement scales. Specifically, SPSS 26.0 was used to assess the internal consistency of the scales, and AMOS 26.0 was employed to assess composite reliability and validity of convergence. Subsequently, to ensure the reliability of the collected data, a common method bias analysis was conducted using SPSS 26.0. Next, descriptive statistical analyses and correlation analyses were performed with SPSS 26.0 to explore the relationships among servant leadership, hindrance stressors, emotional exhaustion, and physical health. Finally, we utilized Model 6 in Process 4.1 software to assess the impact of servant leadership on the physical well-being of elementary school teachers, as well as the sequential chain effects of hindrance stressors and emotional exhaustion on physical health through servant leadership. A self-selected sampling strategy with 5,000 samples was used to obtain a 95% bias-corrected confidence interval (CI) and guarantee robust standard errors. The fit of the proposed model was further assessed using AMOS 26.0, yielding good fit indices: *χ*^2^/df = 3.317, CFI = 0.945, GFI = 0.911, TLI = 0.939, RMSEA = 0.053, and RMR = 0.044.

## 4 Results

### 4.1 Multicollinearity and common methods bias tests

We conducted multicollinearity tests to ensure that the independent variables in the regression model exhibited low intercorrelation. When the tolerance fell below 0.1 or the variance inflation factor (VIF) exceeded 10, it suggested potential multicollinearity among the independent variables. In this study, the tolerance values for each variable ranged between 0.336 and 0.614, whereas the corresponding variance inflation factor (VIF) values varied from 1.629–2.978. These results imply that multicollinearity was not a major issue for the analysis.

To evaluate the potential influence of common method bias in this research, a test for common method bias was performed. Harman’s single-factor test revealed that a single factor explained 43.64% of the variance, well below the 50% threshold, suggesting that no substantial issues with common method bias were evident (87).

### 4.2 Descriptive statistics and correlation analysis

An initial descriptive analysis was conducted to determine the average values and standard deviations of all the variables, along with calculating the Pearson correlation coefficients among them. The results are presented in Table 1. These findings indicate that servant leadership is negatively correlated with hindrance stressors, emotional exhaustion, and physical health. Additionally, hindrance stressors are positively correlated with emotional exhaustion and physical health, and emotional exhaustion is positively correlated with physical health.

**Table 1:** Means, standard deviations and intercorrelations among variables.

Variables	M	SD	1	2	3	4
1. SL	3.296	0.907	1	0.617^**^	−0.527^**^	−0.399^**^
2. HS	3.062	0.874	−0.617^**^	1	0.778^**^	0.534^**^
3. EE	2.978	0.924	−0.527^**^	0.778^**^	1	0.583^**^
4. PH	2.856	0.688	−0.399^**^	0.534^**^	0.583^**^	1

**p < 0.01.

### 4.3 Chain mediation effect test: the influence of servant leadership on physical health

To evaluate the proposed hypotheses, we utilized Hayes’ SPSS PROCESS macro to perform a sequential mediation analysis, using Model 6 to assess the mediating effects. Bootstrap resampling with 5,000 replications was applied to determine the significance of the mediating role. Significance was assessed based on whether the 95% confidence interval (CI) contained zero. Specifically, a significant mediation effect was identified if zero was not included in the CI, whereas the presence of zero within the interval suggested a nonsignificant effect.

**Figure 2:**
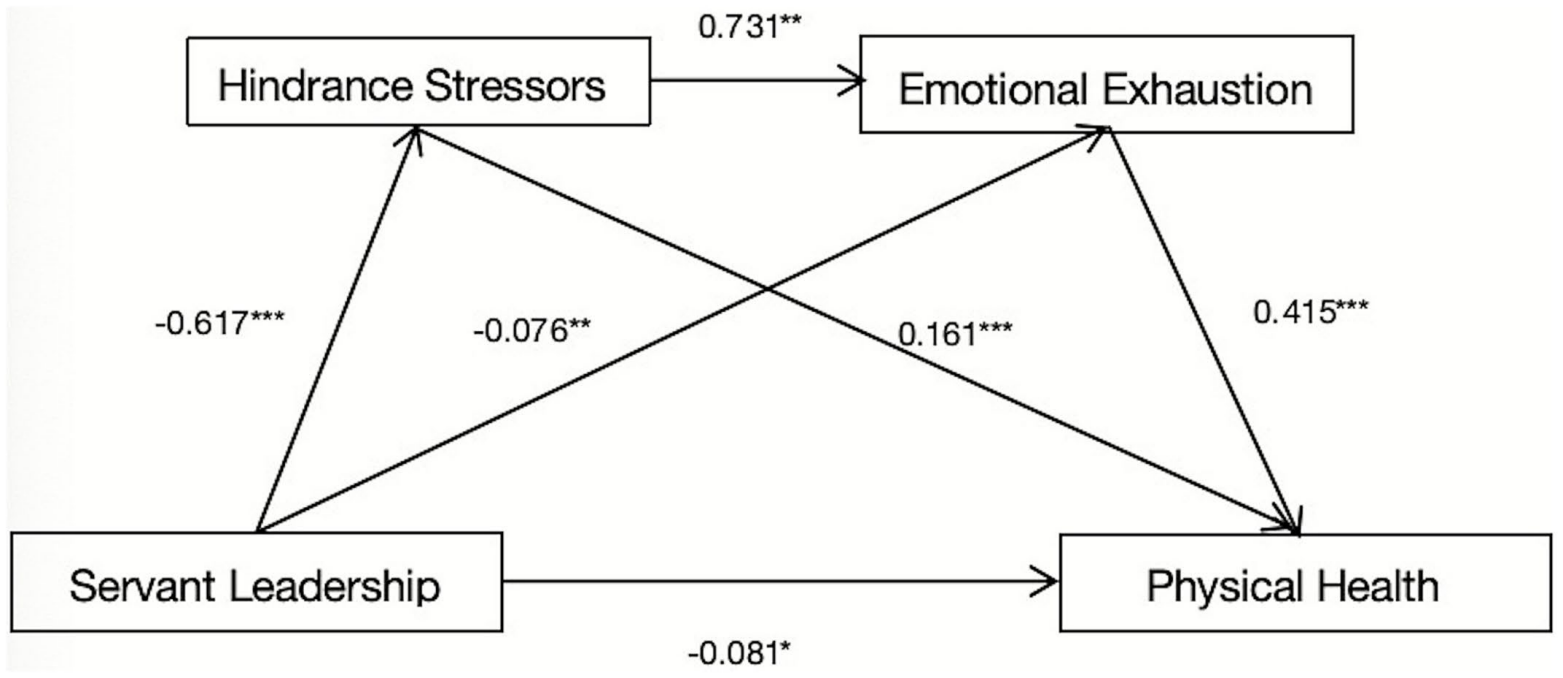
Model diagram of the effect of servant leadership and physical health.

The findings demonstrate (see Table 2; Figure 2) that servant leadership significantly negatively predicts physical health (*β* = −0.399, *p* < 0.001), hindrance stressors (*β* = −0.617, *p* < 0.001), and emotional exhaustion (*β* = −0.076, *p* < 0.01). Additionally, hindrance stressors significantly positively predict emotional exhaustion (*β* = 0.731, *p* < 0.01). When servant leadership, hindrance stressors, and emotional exhaustion were simultaneously included in the model, servant leadership significantly negatively predicts physical health (*β* = −0.081, *p* < 0.05). In contrast, both hindrance stressors and emotional exhaustion positively predict physical health (*β* = 0.161, *p* < 0.001; *β* = 0.415, *p* < 0.001, respectively).

**Table 2:** Regression analysis of the sequential mediation model.

Regression equation variable		Overall fit index of the equation			Significance of regression coefficients		
Outcome variable	Predictor variable	*R*	*R* ^2^	*F*	*β*	SE	*t*
Physical health		0.399	0.159	151.146^***^			
	Servant leadership				−0.399	0.024	−12.496^***^
Hindrance stressors		0.617	0.381	506.654^***^			
	Servant leadership				−0.617	0.026	−22.509^***^
Emotional exhaustion		0.780	0.608	640^***^			
	Servant leadership				−0.076	0.028	−2.748^**^
	Hindrance stressors				0.731	0.029	26.382^**^
Physical health		0.600	0.360	154.341^***^			
	Servant leadership				−0.081	0.027	−2.280^*^
	Hindrance stressors				0.161	0.038	3.338^***^
	Emotional exhaustion				0.415	0.033	9.318^***^

The values of each variable in the model were standardized prior to substitution into the equation. ***p < 0.001; **p < 0.01; *p < 0.05.

According to the results of the mediation analysis (see Table 3), the total indirect effect of servant leadership on physical health is −0.241, accounting for 79.538% of the total effect, whereas the direct effect is −0.062, accounting for 20.462% of the total effect. Servant leadership impacts physical health via three primary pathways: (1) the direct mediation pathway of servant leadership → hindrance stressors → physical health, with an impact value of −0.075 (24.752% of the total effect); (2) the direct mediation pathway of servant leadership → emotional exhaustion → physical health, with an impact value of −0.024 (7.921% of the total effect); and (3) the chain mediation pathway of servant leadership → hindrance stressors → emotional exhaustion → physical health, with an impact value of −0.142 (46.865% of the total effect). Bootstrap analysis revealed that the 95% confidence intervals for all three pathways do not include zero, indicating that servant leadership affects physical health not only through the individual mediating roles of hindrance stressors and emotional exhaustion but also through the sequential mediating effect of these factors.

**Table 3:** Test of the chained mediation model effects.

Path	Efficiency value	SE	95% CI	Percentage of total effect
Total effect	−0.303	0.024	[−0.350, −0.255]	
Total indirect effect	−0.241	0.023	[−0.286, −0.197]	79.538%
Direct effect	−0.062	0.027	[−0.115, −0.009]	20.462%
Servant leadership → hindrance stressors → physical health	−0.075	0.026	[−0.127, −0.026]	24.752%
Servant leadership → emotional exhaustion → physical health	−0.024	0.010	[−0.045, −0.005]	7.921%
Servant leadership → hindrance stressors → emotional exhaustion → physical health	−0.142	0.018	[−0.177, −0.108]	46.865%

## 5 Discussion and implications

### 5.1 Discussion

Based on the JD-R model and the direct effects hypothesis of social support theory, we investigated the pathway through which servant leadership impacts teachers’ physical health and constructed a chain mediation model with hindrance stressors and emotional exhaustion as mediating variables. To confirm the negative predictive effect of servant leadership on teachers’ physical health problems, a questionnaire-based methodology was employed. These findings contribute to efforts to alleviate teachers’ physical health issues and further enrich the research on servant leadership.

This study confirms that physical health issues among teachers are significantly predicted by servant leadership, with stronger servant leadership behaviors associated with fewer physical health issues, thereby supporting Hypothesis 1. These findings align with prior research demonstrating that servant leadership positively and significantly predicts teachers’ general health (50). Theoretically, servant leadership can have a direct impact on teachers’ physical health as a kind of social support, offering fresh evidence in favor of the direct effect hypothesis. This conclusion expands the impact of servant leadership on its followers, extending its positive influence beyond attitudinal and behavioral aspects to the domain of physical health. The ability of servant leadership to directly alleviate teachers’ physical health issues may stem from its role as a form of social support, which can shape health-related behaviors such as smoking, drinking, and seeking medical care. Especially in China, people generally prefer to trust in authority figures (88). The emphasis servant leadership places on employees’ physical well-being in the workplace may subtly influence their lifestyle choices. Another potential mechanism through which servant leadership enhances employees’ physical health lies in its focus on holistic development (89). Employees’ physical well-being is a crucial foundation for overall personal growth. Servant leadership can promote it by providing health-related resources and support.

We verified the mediating role of emotional exhaustion between servant leadership and physical health. Servant leadership can impact teachers’ physical health through its influence on emotional exhaustion, thus confirming the validity of Hypothesis 2. The participants in this study were primary school teachers in China, where individuals tend to suppress their own needs and emotions (90). However, long-term suppression of one’s emotions may exacerbate or prolong a person’s negative emotions, ultimately reducing their emotional well-being (91). Fortunately, the qualities of leadership might significantly influence teachers’ choices regarding emotion regulation strategies. On the one hand, servant leadership tends to encourage teachers to use more positive emotion self-regulation strategies, such as cognitive reappraisal (46). On the other hand, by fostering understanding, support, and a positive work environment, servant leadership may enhance the confidence of primary school teachers in expressing their needs, seeking more support and finding emotional relief. Additionally, emotional exhaustion can significantly positively predict physical health, indicating that higher levels of emotional exhaustion are associated with more severe physical health problems (8, 62).

Servant leadership influences teachers’ physical health by reducing hindrance stressors, thereby supporting Hypothesis 3. These findings align with those reported in previous studies (80), reinforcing the role of leadership in mitigating workplace stressors. Similarly, Fernet et al. (76) reported that transformational leadership reduces job demands, whereas Bakker and Demerouti (71) highlighted how other leadership styles impact job demands. By showing that servant leadership can also result in fewer job demands, our study fills that gap. The JD-R paradigm, which takes a top-down approach to organizational job design, can be used to explain this occurrence. According to the model, management and human resource departments influence the work environment through task goal setting and resource allocation, thus affecting employees’ exposure to stressors (71). Servant leadership, which prioritizes employee well-being and needs, is vital in this process. Several reasons might explain why servant leadership is negatively correlated with hindering stressors. First, China’s principal responsibility system centralizes authority in the hands of school principals to ensure unified decision-making. As a result, in China, a principal’s leadership style directly influences school administrative efficiency, the management team’s approach, the overall work atmosphere, and interpersonal relationships. Second, the participants in this study are ordinary primary school teachers who are at the lower end of the school management hierarchy (92). Servant leaders exhibit strong support by prioritizing employees’ needs, fostering their growth, and offering them necessary assistance, which may help alleviate the hindrance stressors caused by insufficient resources and challenging conditions for task completion. Additionally, hindrance stressors are significantly positively correlated with physical health problems. This conclusion is consistent with the findings of existing studies that posit that hindrance stressors are positively correlated with physical health problems (67, 68).

This research clarified the mediating roles played by hindrance stressors and emotional exhaustion in the relationship between servant leadership and physical health, thereby confirming Hypothesis H4. The finding that hindrance stress predicts emotional exhaustion aligns with prior research (81) and supports a key principle of the challenge–hindrance stressor framework (CHSF), which suggests that hindrance stressors are generally linked to adverse outcomes, including burnout and mental health issues (65). In educational contexts, hindrance stress refers to the pressures that hinder teachers from completing their work tasks and achieving professional development. These may encompass role ambiguity, inadequate resources for professional advancement, onerous administrative regulations, substantial non-teaching responsibilities, and interpersonal conflicts among colleagues. Servant leadership values teachers’ growth and needs and takes measures such as assigning clear and feasible tasks, providing sufficient resources for professional development, reducing administrative burdens, minimizing bureaucratic constraints, and promoting cooperation among teachers to reduce hindrance stress in the work environment. The reduction of hindrance stress enables teachers to avoid continuously expending extra energy to cope with unnecessary pressures, thereby decreasing the depletion of psychological resources, reducing emotional exhaustion, and mitigating health problems caused by emotional exhaustion. According to JD-R theory, emotional exhaustion and physical health problems are both health issues triggered by job demands. The alleviation of emotional exhaustion can relieve psychological stress, reduce physiological stress responses, and encourage teachers to adopt healthier behavioral patterns, thereby improving their physical health. This study further refines the internal structure and relationships of these health issues to promote further investigation into their fundamental processes and operational paths.

### 5.2 Implications

This study contributes to the literature on employee health and leadership in several key ways. First, although physical health issues can adversely affect both individuals and businesses, current research has focused predominantly on the effects of leadership on employees’ mental health, with minimal investigations into its impact on physical health. By addressing this gap, this study provides empirical support for the direct effect hypothesis of social support theory and deepens our understanding of how leadership affects employee physical health. Second, this study empirically confirms the relationships between servant leadership, hindrance stressors, emotional exhaustion, and physical health. It reinforces the claim that leadership can shape employee well-being by influencing job demands (71), thereby offering a potential avenue for advancing JD-R theory. Specifically, the findings establish a negative correlation between servant leadership and hindrance stressors, as well as the direct impact of servant leadership on teachers’ emotional exhaustion and physical health. These discoveries could stimulate further study on leadership and employee well-being in many cultural and industrial situations, thereby broadening the theoretical and practical ramifications of leadership studies.

These findings also have important practical implications regarding Chinese elementary teachers’ physical and mental well-being. First, school administrators should emphasize teachers’ physical health and prioritize teachers’ physical well-being by implementing stress management strategies, advocating for healthy lifestyles, and offering guidance to enhance teachers’ overall health. Specifically, school leaders can provide teachers with additional health education through expert lectures and social media dissemination and include outdoor physical activities at teachers’ union events to encourage regular exercise and reduce physical strain. Second, schools should implement a form of servant leadership that focuses on teachers’ emotional health, emphasizes empathy and compassion, establishes positive psychological connections with teachers, and encourages teachers to adopt positive emotional regulation measures. Moreover, school leaders should provide greater humanistic care to teachers by establishing effective mechanisms for collecting and responding to teachers’ opinions. It is essential to create open communication channels that allow teachers to express their concerns and emotional challenges in a supportive environment. In addition, administrators should identify the real problems that affect teachers’ well-being and take timely actions to address their practical needs and challenges. Third, this study highlights the fact that servant leadership reduces teachers’ emotional exhaustion and physical health issues by decreasing hindrance stressors. Servant leadership that directly reduces hindrance stressors is more efficient than intervening after the emergence of hindrance stressors. School administrators should increase their administrative effectiveness and leadership skills to create a work environment with low hindrance stressors, thereby enhancing teachers’ physical health. From an administrative perspective, education authorities should take active measures to organize professional training programs for school principals, aiming to enhance their management competence and strengthen their capacity to create supportive, harmonious, and efficient working environments for teachers.

### 5.3 Research limitations and future prospects

This research has several limitations. First, the reliance on self-report scales for variable assessment may introduce biases, which could compromise the accuracy of the results. Future research should consider incorporating more objective measurement approaches, such as physiological indicators or third-party evaluations (93). Second, the cross-sectional research method restricts the ability to infer causal relationships. Future studies should utilize longitudinal or experimental designs to clarify causal relationships. Third, the gender imbalance among respondents may result in certain sampling bias, which could affect the generalizability of the study. In future studies, more balanced sampling strategies will be adopted to improve the representativeness of the gender distribution.

## 6 Conclusion

This study examined the impact of servant leadership on teachers’ physical health and explored the mediating roles of emotional exhaustion and hindrance stressors in this relationship. The findings indicate that perceived servant leadership is negatively correlated with teachers’ physical health problems, hindrance stressors, and emotional exhaustion. In contrast, hindrance stressors are positively associated with both emotional exhaustion and physical health issues, and emotional exhaustion further contributes to negative physical health outcomes. Moreover, the results of this study confirm that hindrance stressors and emotional exhaustion serve as mediating mechanisms through which servant leadership influences teachers’ physical health.

## Data Availability

The raw data supporting the conclusions of this article will be made available by the authors, without undue reservation.
